# The network of commodity risk

**DOI:** 10.1007/s12667-022-00530-7

**Published:** 2022-08-20

**Authors:** Beatrice Foroni, Giacomo Morelli, Lea Petrella

**Affiliations:** 1 MEMOTEF Department, Sapienza University of Rome, 00161 Rome, Italy; 2 Department of Statistical Sciences, Sapienza University of Rome, 00185 Rome, Italy

**Keywords:** Financialization, Commodity markets, Value-at-Risk, Graphical LASSO, C44, C45, C51, C54, Q02

## Abstract

In this paper, we investigate the interconnections among and within the Energy, Agricultural, and Metal commodities, operating in a risk management framework with a twofold goal. First, we estimate the Value-at-Risk (VaR) employing GARCH and Markov-switching GARCH models with different error term distributions. The use of such models allows us to take into account well-known stylized facts shown in the time series of commodities as well as possible regime changes in their conditional variance dynamics. We rely on backtesting procedures to select the best model for each commodity. Second, we estimate the sparse Gaussian Graphical model of commodities exploiting the Graphical LASSO (GLASSO) methodology to detect the most relevant conditional dependence structure among and within the sectors. A novel feature of our framework is that GLASSO estimation is achieved exploring the precision matrix of the multivariate Gaussian distribution obtained using a Gaussian copula with marginals given by the residuals of the aforementioned selected models. We apply our approach to the sample of twenty-four series of commodity futures prices over the years 2005–2022. We find that Soybean Oil, Cotton, and Coffee represent the major sources of propagation of financial distress in commodity markets while Gold, Natural Gas UK, and Heating Oil are depicted as safe-haven commodities. The impact of Covid-19 is reflected in increased heterogeneity, as captured by the strongest relationships between commodities belonging to the same commodity sector and by weakened inter-sectorial connections. This finding suggests that connectedness does not always increase in response to crisis events.

## Introduction

The financialization of commodities [16, 28, 100] has drawn the attention of risk managers and financial institutions on the propagation of the commodity risk that arises from the fluctuations of commodity future price values [70, 79]. Commodities are actively traded in financial markets and have been largely used for hedging purposes [73]. However, market volatility makes commodity prices vulnerable to highly correlated shocks [41] creating significant business challenges that affect financial performances, exacerbate well-known spillovers effects among commodities, and tight credit availability.

Hidden severe consequences affect the economic system as well, especially in those countries where commodities are heavily employed as raw materials (Crude Oil, Gasoline, Natural Gas, Copper, Aluminium, and agricultural commodities) in the industrial sector. Indeed, UNCTAD [109] reports that over the last two decades 67% of developing countries has been relying on commodities, a percentage that rises to 80% when considering only the least developed countries. Therefore, a big concern for risk managers and policy-makers becomes the monitoring of the propagation of the commodity risk in commodity markets which requires the development of new operational approaches [8, 17, 58]. The understanding of the propagation of the commodity risk through the financialization of commodities requires to be detected designing a framework that accounts for the specific contribution of the single commodities to the market risk. This is the aim of our work.

The relevance of the issue is particularly highlighted by the role of the commodity risk within the regulatory framework. The Basel Accords establish a minimum capital standard to cover the risk of holding or taking positions in commodities and impose each bank subject to capital charges for market risk to monitor and report the level of commodity risk against which a capital requirement is to be applied [15]. Over the past decades, rich literature has flourished to propose valuable instruments for measuring and quantifying such risk. The most employed market risk measure is the Value-at-Risk (VaR), defined as the worst expected loss of an asset or a portfolio given a certain confidence level and over a specific time period [71]. It is a crucial component of risk management when designing and monitoring an appropriate modelling framework able to quantify commodity price risk exposure, avoiding unexpected large losses [8].

In this paper, we propose a framework with a twofold risk management goal: i) forecasting commodity risks and spillovers to identify and understand the factors that drive commodity markets and ii) capturing the impact of contagion of such risk on the stability of the financial system. We combine an econometric and statistical set-up where several models are compared and backtested to find the one that better estimates the VaR for each commodity futures returns. The network is built on the residuals of the models chosen according to a risk management approach. The network approach guides decision-makers in the field through the investigation of the extent to which uncertainty in commodity prices affects the practical transmissions of the commodity risks [88].

More specifically, we carry out the first task estimating the VaR of the commodities through GARCH and Markov-switching GARCH (MS-GARCH) models with different distribution of the innovations. The choice of GARCH-type models accommodates the typical stylized facts of commodity time series such as volatility clustering, skewness, kurtosis [1, 37, 38, 110], and regime changes in the conditional variance dynamics. We perform model selection relying on targeted tests procedures and evaluate the model that outperforms the others from a risk management point of view, i.e. from a VaR forecasting perspective [76]. To do this, we use three backtesting procedures: the Unconditional Coverage (UC) test of Kupiec [75], the Conditional Coverage (CC) test of Christoffersen [32], and the Dynamic Quantile (DQ) test of Engle and Manganelli [45]. We refer to Masala [80] for a recent survey about the role of backtesting procedures in commodity portfolios. To contemplate the stylized facts of commodity returns, we consider various specifications in the model selection procedure such as Normal GARCH, Skewed Normal GARCH, Student’s-t GARCH, Skewed Student’s-t GARCH, Generalized Error GARCH, Skewed Generalized Error GARCH, Normal MS-GARCH, Skewed Normal MS-GARCH, Student’s-t MS-GARCH, Skewed Student’s-t MS-GARCH, Generalized Error MS-GARCH, Skewed Generalized Error MS-GARCH, thus empowering our framework with the flexibility that the policy-maker needs.

The second task deals with the estimation of the interconnections among and within the commodity sectors considered. To explore this issue we use a graphical model approach which is an intuitive way of representing and visualizing the relationships among many variables. In particular, to manage the interconnection structure of commodity markets, we exploit the Graphical LASSO (GLASSO) methodology proposed by Friedman et al. [54] to estimate a sparse Gaussian Graphical model and detect the strongest conditional dependencies among the commodities. Since GLASSO relies on the assumption of gaussianity, we build a Gaussian copula with marginals obtained from the residuals of the best model evaluated under the backtesting criteria. Moreover, to synthesize the information contained in the graphical model, we compute the eigenvector centrality measure that shows the most relevant commodities within the network structure in terms of the influence of each node in the graph. This allows us to guide decision makers [88] ranking the commodities in the network according to their importance in the propagation of the commodity risk.

We collect the future prices of twenty-four commodities belonging to the commodity sectors Agriculture, Energy, and Metals over the sample period that spans from October 3, 2005 to March 25, 2022. Our findings show that the MS-GARCH outperforms GARCH models in 75% and 58% of the cases before and after the Covid-19 pandemic, respectively. From a graphical point of view, the network analysis reveals that commodities are overall densely connected and the Covid-19 shock hits such connectedness reducing the density of the graph by 18%. The degree of connectedness between the commodity sectors is also affected. Inter-sectorial linkages are severely weakened as opposed to those among commodities within the same sectors. In fact, before Covid-19 Coffee and Soybean Oil present the maximum rate of connections with the Energy sector coherently with Myint and El-Halwagi [82], Dunis et al. [44], and Al-Maadid et al. [3] where the spillover effect between Coffee and the energy sector results even increased after the global financial crisis in 2008. With the outbreak of Covid-19, these patterns have been disentangled. Concerning the specific roles of the commodities in the network, Soybean Oil occupies the most central node in the graph only after the pandemic while Natural Gas was the most connected until 2018 (in line with Ergen and Rizvanoghlu [46]). Remarkable changes are also encountered in the safe-haven commodities. Before Covid-19, they were Heating Oil, Soybean Meal, and Gold and after the pandemic, they become Natural Gas UK, Gold, and Heating Oil.

In a similar spirit to our paper, other studies have investigated commodity connectedness [12, 41, 115]. However, their modelling frameworks are characterized by a common distributional assumption for the commodities included in the analysis and do not simultaneously address the assessment of the market risk. Indeed, the novelty of our approach lies in the combination of backtesting procedures, Gaussian Copula, and GLASSO estimation approach for the network. Unlike traditional network estimation based on the single assumption on the vector of observations, we build the network of commodity risk basing on the residuals of GARCH-type models with underlying distribution selected for each commodity through the backtesting procedures thus accommodating for well-known stylized facts. The dependence structure of the residuals is captured by the Gaussian Copula. Centrality measures retrieved from the sparse estimated network are exploited to guide decision makers choices through commodity risk management. To the best of our knowledge, this is the first paper that addresses this issue.

The rest of the paper is organized as follows. Section 2 discusses the major strands of the literature to which this paper contributes. Section 3 provides a brief outline of the employment of the GARCH-type models and the backtesting procedures for model selection. Section 4 presents the GLASSO model. Empirical results are reported in Sect. 5 and the conclusions are in Sect. 6.

## Literature review

The effects of commodity price fluctuations on the macro-economy have been pioneered in Hamilton [62], and the connection with economic growth has been a fruitful thematic in the financial literature [18, 21, 24, 25, 27, 40, 74, 108, 112, 114]. Despite the key informational role of commodity futures in addressing the monetary policy [9, 66] the exposure of commodity price fluctuations to macro risk has been hard to price [95] due to the strong inter-sectorial dependencies, first documented in Pindyck and Rotemberg [90] and Ciner [35]. A rich literature has then focused on the spillover effects between oil price and financial markets [72], and oil price and other commodities [10, 39] such as precious metals [47, 93], agricultural [43, 84, 85], energy [49, 92, 102], and, more recently, the impact of climate related variables on the co-movements of commodity prices that affect the stability of the financial system [50]. Besides, commodity price behavior shows small trends and big variability that affects market preferences also in the long-run [23, 30, 103].

Spillovers effects have particularly intensified since 2004, the onset of the financialization of commodity markets [16, 28, 65, 100]. From that moment onward, commodities have been considered among the likely sources of financial distress due to the centrality of the role acquired. Gradually, they have revealed to be responsive to macro-economic shocks and to investors sentiment [57, 91, 98, 105] and to be strongly connected to widely spread financial instruments [77, 83, 113].

Major operational and management implications caused by the financialization of commodities spring up in the uncertainty of the decision-making processes for the related industries. Examples concern the role of supporting the management of refinery operations and productions of oil and gas, or the management generator operations and the supply chain due to the non-storable nature of the production of energy, as discussed in Andriosopoulos and Nomikos [6], Gabrel et al. [55], Joëts [70], Aven [8], and Nguyen et al. [88].

Such relevance of commodities from various perspectives of the financial system has encouraged the assessment of the market risk for commodity markets (see, among others, [89]). Giot and Laurent [59] introduce the use of VaR to compute the contribution of the commodities to the market risk and find that for market participants trading short positions the risk arises from an increase in commodity prices, while for long positions it is given by a price drop. Marimoutou et al. [78] apply VaR to the oil market and Aloui and Mabrouk [5] study VaR estimations of energy commodities documenting financial stylized facts such as long-memory, asymmetry, and fat tails. Laporta et al. [76] investigate the selection of VaR for energy commodities, whereas Algieri and Leccadito [4] and Ji et al. [68] propose a (Co)VaR based model to study risk spillovers between energy and non-energy commodity markets. Shen et al. [97] integrate VaR estimation in a Vector Autoregression (VAR) to evaluate the risk transmission channel in energy markets. An alternative approach to address systemic risk in a VaR set-up is through copula [81]. VaR forecasting may help risk managers and regulators to evaluate the exposure to unexpected loss and consequently calibrate the overall riskiness of financial markets.

Besides VaR computation, many studies have delved into the analysis of the connectedness among commodity markets [12, 41, 106, 107] and between commodity and financial markets. Ji and Fan [69] propose a graph analysis of the evolution of the world crude oil market whereas the works of Diebold et al. [41], Zhang and Broadstock [115], and Balli et al. [12] derive the connectedness of commodities from the Diebold and Yılmaz [42] forecast-error variance decomposition matrix of a vector autoregressive (VAR) model. In Diebold et al. [41], the VAR is constructed from the range-based realized volatility of Garman and Klass [56], whereas in Balli et al. [12] it comes from the commodity uncertainty index proposed in Chuliá et al. [34] that builds on the residuals of a generalized dynamic factor model.

These studies have tackled several relevant issues in the analysis of the mechanisms of commodity markets.^1^ However, the major research questions concerning the financialization of commodities, commodity connectedness, and the assessment of the market risk remain separately addressed. In particular, in the field of the study of commodity connectedness, the modelling framework of the previous literature exploits common distributional assumptions for the commodities while commodity risk management requires models that account for the structural differences between the commodities to gauge the different risk exposures. Hence, for a robust detection of the major risk transmitters in commodity markets, methodologies must be integrated with new approaches that consider the stylized facts of the single commodities. For this reason, we propose a framework that merges commodity connectedness and modelling selection according to market risk criteria.

## Model specifications

It is well-known that the time series of commodities show most of the stylized facts detected in financial markets such as skewness, kurtosis, and volatility clustering. Moreover, recent studies have shown that the variance process often exhibits regime changes [61] and that ignoring this feature affects the precision of the volatility forecast [36].

Throughout the paper, we consider the GARCH(1,1) and MS-GARCH(1,1) models with different conditional distribution ${\mathcal {D}}_{\Theta }(\cdot )$ to account for stylized facts. Overall, our framework includes 12 model specifications, recovered as a combination of:

- the conditional variance specification: GARCH(1, 1) and MS-GARCH(1,1);
- the choice of the conditional distribution ${\mathcal {D}}_{\Theta } \in$ $\lbrace$norm, snorm, std, sstd, ged, sged$\rbrace$.

We detail the description of the model specification in the Appendix. The choice of the conditional variance specifications are supported by the works of Bollerslev et al. [20], Sadorsky [96], Huang et al. [67] and, especially, Hansen and Lunde [63]. Moreover, GARCH(1,1) models have proved good fitting performance on commodities [76]. We remark that the specifications considered are only intended to provide an example of design of policy for commodity markets that simultaneously addresses the commodity interdependencies and the exposure to the market risk. Among the conditional distributions, we include the standardized skewed version of each model implemented via the Fernández and Steel [48] transformation. We denote the standardized Skewed Normal, the Skewed Student’s-t, and the Skewed Generalized Error Distribution by "snorm", "sstd", and "sged", respectively. For the MS-GARCH specification, we focus on double-regime MS-GARCH models accounting for low and high volatility levels, thus the scale and asymmetry parameters vary with the regimes.

In line with the first goal of the paper, we consider a wide range of models and use a risk management approach that selects the best models to accurately predict future risks, especially in the case of volatility models [33]. We select the model that provides the most reliable forecast of the VaR performing backtests [31].

## Sparse Gaussian graphical model

The second goal of the paper is to study the interdependence among and within the commodity sectors. To achieve this purpose, we rely on the Gaussian Graphical Lasso (GLASSO) methodology proposed in Friedman et al. [54]. GLASSO allows us to build an undirected Gaussian graphical model and perform a network representation of the connections of the *commodity risks* where only the most relevant intra- and inter- sectorial linkages are highlighted. This is conveniently accomplished estimating a sparse conditional dependence structure among the commodities. That is, we estimate the inverse Gaussian covariance matrix, $\Omega = \Sigma ^{-1}$, where the zero off-diagonal elements correspond to a pair of commodities returns that are conditionally independent [64]. More specifically, GLASSO builds on Tibshirani [101] where a penalized maximum likelihood problem shrinks to zero some coefficients through a $L_1$-norm penalty term as follows:

1 $$\begin{aligned} \Omega ^{*}=\text {arg max}_{\Omega } \log (\det \Omega - tr(\Sigma \Omega ) - \rho \left\| \Omega \right\| _1), \end{aligned}$$

where $tr(\cdot )$ denotes the trace operator and $\left\| \Omega \right\| _1$ the $L_1$-norm that can be calculated as the sum of the absolute values of the elements of $\Omega$. The parameter $\rho$ controls for the size of the penalty and it determines the number of zeros in the sparse precision matrix $\Omega$: a higher (lower) value is responsible for a more (less) sparse matrix. Like most of the shrinking methodologies, the right choice of the penalization parameter $\rho$ is fundamental to obtain a reliable selection. To estimate the optimal value of $\rho$, we minimize the Extended Bayesian Information Criterion (EBIC, [26]) which has been shown to work particularly well in retrieving the true network structure [14, 53] and it is a computationally efficient alternative to cross-validation [111]. The criterion is indexed by the hyperparameter $\gamma \in [0,1]$. Typical values of $\gamma$ are 0, 0.5 or 1 with values closer to 1 leading a stronger penalization. For this reason, in the empirical application of this paper we choose $\gamma = 1$.

As stated above, GLASSO relies on the assumption of Gaussianity introduced through the Gaussian copula. In particular, the copula approach provides the framework to model multivariate associations from the univariate distributions of the observed variables. In the case of the *d*–random vector, $X=(X_1,\dots ,X_i,\dots ,X_d)$, with marginal cumulative distributions $F_i(x_i)=P(X_i<x_i)$, we can define the joint cumulative distribution function (cdf) as $F(x)=P(\cap _{i=1}^d X_i<x_i)$. In many cases, the margins of the cdf are relatively easy to describe, but an explicit expression of the joint distribution may be difficult to obtain. When $X \sim N_d(\mu , \Sigma )$ is a Gaussian random vector then its copula is called Gaussian copula. Denoting $u_i \equiv F_i(x_i)$, the Gaussian copula is defined by the cdf $C^{Ga} (u_1,\dots ,u_i,\dots ,u_d)= P(\cap _{i=1}^d \Phi (X_i) \le u_i) = \mathbf {\Phi } (\cap _{i=1}^d \Phi ^{-1}(u_i))$ where $\Phi ^{-1}(\cdot )$ is the univariate standard Gaussian quantile function and $\mathbf {\Phi }(\cdot )$ is the *d*-variate Gaussian cdf with mean 0 and covariance matrix $\Sigma$.

### Network metrics

Network metrics are used to synthesize the information contained in a graphical model. In this section, we briefly introduce the definitions and the metrics that we use to detect the position of a commodity within the network.

We define an undirected graph as an ordered pair of two disjoint sets (*V*, *E*), where *V* is the set of vertices and *E* is the set of edges, consisting of pair of elements taken from *V*. We denote the number of vertices with $n = |V|$ and the number of edges with $m=|E|$. The density, *D*, of the graph is given by the ratio between the number of edges and the number of possible edges:

2 $$\begin{aligned} D = \frac{2m}{n(n-1)}. \ \ \ \end{aligned}$$

Vertices *i* and *j* are adjacent if the undirected edge between *i* and *j* is in the set *E*, and a line connects them in the diagram of the graph. The matrix representation of such a graph is obtained via the adjacency matrix, $A_G$, of the inverse covariance matrix, $\Omega$. The single element in $A_G=(a_{ij})$ is $a_{ij}=1$ if the corresponding element of the inverse covariance matrix is positive, $a_{ij}=0$ if the corresponding element is zero. Hence, the graph contains an edge that links two vertices *i* and *j* if and only if $a_{ij}=1$. A simple yet fundamental metric is the degree of a node defined by $k_i = \sum _j a_{ij}$, which measures the number of neighbors of the node.

Centrality measures are crucial metrics used in the network topology to highlight nodes that occupy critical positions in the graph. For instance, the eigenvector centrality, or Gould’s index of accessibility [60], indicates which are the most geographically central and important nodes, and it has been exploited in financial applications to capture the capacity of an agent to cause systemic risk [19]. It builds on the eigenvector assigned to the leading eigenvalue of the adjacency matrix to assign a relative score to the nodes, depending on how connected they are to the rest of the network. A metric that is strictly related to the eigenvector measure is the eigenvector community structure [87], which allows us to create a subgraph starting from a group of vertices densely connected, linked with other groups of vertices through sparse connections. The eigenvector community structure depends on the spectrum of the modularity matrix *B*, with elements defined as:

3 $$\begin{aligned} b_{ij}=a_{ij} - \frac{k_i k_j}{2m}. \ \ \ \end{aligned}$$

$a_{ij}$ are the elements of the adjacency matrix and $\frac{k_i k_j}{2m}$ is the number of edges between vertices *i* and *j* if edges are placed at random, where $k_i$ and $k_j$ are the degrees of the vertices, and $m = \frac{1}{2}\sum _i k_i$ is the total number of edges in the network. The algorithm to compute the eigenvector community structure calculates the leading eigenvector of the *modularity matrix* and divides the vertices into two groups according to the signs of the elements in this vector. The values of the leading eigenvector assess the importance of each vertex in its community: a larger (smaller) value corresponds to a more (less) central member.

## Empirical results

### Data description

We collect data of twenty-four time series of commodity futures prices from Bloomberg over the period that spans from October 3, 2005 to March 25, 2022 for a total of 4300 observations. The commodities in the sample belong to the commodity sectors Agriculture, Energy, and Metals and are divided as follows:

- Agriculture: Coffee (KC1), Oats (O1), Soybeans (S1), Wheat (W1), Cocoa (CC1), Corn (C1), Rough Rice (RR1), Cotton (CT1), Sugar (SB1), Soybean Oil (BO1), Soybean Meal (SM1), and Orange Juice (JO1).
- Energy: Gasoline (XB1), Heating Oil (HO1), Low Sulfur Gasolio (QS1), Natural Gas (NG1), Ethanol (DL1), WTI Crude Oil (CL1), and Natural Gas UK (FN1).
- Metals: Gold (GC1), Silver (SI1), Palladium (PA1), Copper (HG1), and Zinc (LX1)

Daily returns with continuous compounding are calculated taking the logarithm of the difference between closing prices in consecutive trading days and then multiplied by 100.^2^

In Table 3 we report the summary statistics of the log-returns of the commodities as in Laporta et al. [76] before and after the outbreak of Covid-19. The distribution of the returns for each commodity displays fat tails and serial correlation. The Jarque–Bera test significantly rejects the normality behaviour of daily returns, the ARCH Lagrange Multiplier and the Augmented Dickey–Fuller tests suggest the presence of autoregressive conditional heteroskedasticity and the absence of unit roots, respectively. Ethanol and Natural Gas are the only commodities with negative average returns and show high standard deviation. The maximum, however, is reached by WTI Crude Oil with 20.7. The distributions of the commodity returns generally exhibit negative skewness with Natural Gas, Natural Gas UK, Wheat, and Coffee as the only exceptions. The returns of these four commodities are then featured by extreme positive values. The impact of Covid-19 is captured in the higher kurtosis and standard deviation which are amplified in response to the growing level of uncertainty in the market. Besides, the distribution of the returns of the commodities tends to be more negatively skewed indicating the stronger propensity to undergo high losses.

### Backtesting results

We perform backtests over 2699 observations using 1600 days as estimation window. The width of the rolling window allows us to obtain significant estimates of the parameters while providing a trustworthy picture of the fluctuations in the market. Also, an amplitude of six years is sufficient to capture the evolution and major regime changes in the returns. In particular, we include the entire period of the recession in the United States that began in December 2007 and ended in June 2009 according to the National Bureau of Economic Research.^3^

For each time series of commodities, we fit GARCH and MS-GARCH with conditional distribution ${\mathcal {D}}_{\Theta } \in$ $\lbrace$norm, snorm, std, sstd, ged, sged$\rbrace$. The computational analysis is conducted using the software *R* and the package "MSGARCH". We point out that the introduction of GARCH models is only intended to accommodate the different behaviors of commodity returns within a risk management perspective based on the VaR predictability of the model.

We choose the best model out of the 12 models considered according to the backtesting procedures described in the Appendix. We backtest each GARCH-type estimated VaR using the Unconditional Coverage (UC) test of Kupiec [75], the Conditional Coverage (CC) test of Christoffersen [32], and the Dynamic Quantile (DQ) test of Engle and Manganelli [45]. We are concerned with the downside risk at two confidence levels: 95% and 99%. The best models are selected choosing among those that show a p-value higher than the 5% significance level in at least two out of three tests. To determine the final model, we pick the one with the highest p-value relative to the DQ test on the 95-th quantile level. This choice is motivated by a trade-off between the risk management perspective and the need to have an adequate number of observed violations of the estimated quantiles.

Backtesting results are represented in Tables 5, 6, and 7. Overall, many of the model specifications succeed in forecasting the returns volatility of the commodities. On the one hand, we find no prevalence for the asymmetric distributions over the symmetric ones. On the other hand, we detect a considerable prevalence of the Markov-switching specification which feature 14 commodities out of 24 (Table 1). This result contributes, among others, to the findings in Bulla and Bulla [22] and Ardia et al. [7], that show that the Markov-switching specification better captures the breaks in the dynamics of the volatility of financial returns. The effect of Covid-19 is found in the reduced number of optimal MS-GARCH models. Thus, the shock of the pandemic has affected the propensity of regime changes of the volatility of the commodities to describe the evolution of VaR exceedances.

**Table 1:** Best models for the commodities selected according to the backtesting criteria

Commodity	Distribution	Regime
Gold	GED	MS-GARCH
Silver	Student’s-t	MS-GARCH
Copper	Skewed GED	MS-GARCH
Palladium	Student’s-t	MS-GARCH
Zinc	Gaussian	GARCH
WTI Crude Oil	Student’s-t	MS-GARCH
Heating Oil	Skewed Gaussian	GARCH
Low Sulfur Gasolio	Skewed GED	MS-GARCH
Natural Gas	Skewed Student-t	GARCH
Gasoline	Gaussian	GARCH
Natural Gas UK	GED	GARCH
Ethanol	Skewed Gaussian	MS-GARCH
Corn	Student’s-t	MS-GARCH
Oats	Student’s-t	MS-GARCH
Rough Rice	Student’s-t	MS-GARCH
Soybeans	Student’s-t	GARCH
Wheat	Skewed Gaussian	MS-GARCH
Cocoa	Student’s-t	GARCH
Cotton	GED	GARCH
Coffee	GED	GARCH
Sugar	Gaussian	MS-GARCH
Soybean Oil	Gaussian	MS-GARCH
Soybean Meal	Skewed Student’s-t	MS-GARCH
Orange Juice	Student’s-t	GARCH

Sample period: October 3, 2010–March 25, 2022

### The network of commodity risks

**Fig. 1:**
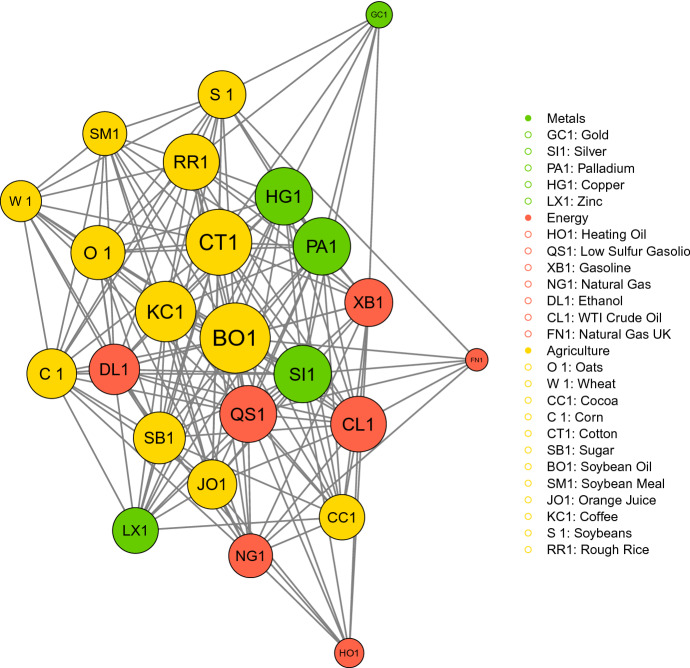
Sparse Gaussian graphical model built on the residuals following the distribution selected according to the backtesting criteria and then aggregated into a Gaussian copula. The optimal tuning parameter for the implementation of the GLASSO is 0.0158. The size of each vertex is proportional to the corresponding Eigenvector Centrality coefficient. Sample period: October 3, 2005–March 25, 2022

We turn the analysis to the estimation and discussion of the network structure among the commodities in the sample. To achieve the estimation of the graph, we fit the Gaussian Copula on the residuals obtained from the GARCH-type model selected according to the the backtesting results. Therefore, the marginals of the Gaussian Copula are given by the distribution of each series of residuals. Then, we estimate the tuning parameter for the GLASSO minimizing the EBIC. To quantify the degree of connectedness within the graph and among specific underlying clusters, we compute the network metrics described in Sect. 4.1. Figure 1 shows *the network of commodity risks*. The computational analysis is conducted using the software *R* and the package "GLASSO". The graph is estimated using the optimal tuning parameter $\rho = 0.0158$ and the size of the nodes in Fig. 1 is proportional to the eigenvector centrality score. The density $D = 0.61$ highlights the strong interconnections in the network. This result is explained in part because of the well-known spillover effects [43, 85] originated from the dependence that links commodities to cycles of production and consumption, and in part because of the effects of the financialization of commodities.

The graphical representation of the results has brought relevant information to the analysis of the network of commodity risk, making easy the interpretation of results, and strengthening previous literature in this field. For instance, the safe-haven role of Gold is immediately captured. In fact, it represents one of the least central nodes in the network. This implies that it has a poor dependence relation with the other commodities and therefore it can be a good investment in anticipation of high volatility periods. Copper is the first central metal commodity in the graph. It is a cheap and plentiful metal, among the most traded in the world markets, and in the years has seen its consumption rise, especially because of building construction and electronic products. Palladium has seen its consumption rise too. Their centrality, together with that of Coffee, provides useful information to investors in the financial markets and serves as a barometer for the stability of the financial markets of large parts of Asia and South America.

In Table 8 we report the relative connections for each commodity defined as the ratio between the number of active connections that the commodity has with a certain sector and the maximum number of possible connections with that sector. For example, Gold has 3 connections with the Metal sector, dividing by the maximum number of possible connections of the metal sector, i.e. 4, we get the value of 0.75. Most of the commodities exhibit the highest connection with the corresponding sector. In particular, this is the case of Copper and Palladium and Silver for the Metal sector, WTI Crude Oil, Low Sulfur Gasoil, and Natural Gas for the Energy sector. It is interesting to notice that some commodity shows particular rates of connection with other sectors. It is worth noting that some commodity show particular rates of connection with other sectors. For example, in the Metals sector, Palladium reports that connection rate of 0.75 with the Agriculture sector. Regarding the Energy sector, Low Sulphur Gasolio shows the maximum rate of connection with the Metals sector (0.80). Concerning the Agricultural sector, Oats, Cocoa, Coffee, and Soybean Oil have high connection rates with other sectors. The high rate of connections among Coffee and the Energy sector is coherent with Al-Maadid et al. [3] where the authors find the spillover effect between Coffee and the Energy sector to be even increased during the post-crisis period. Soybean Oil presents the maximum connection rate with the Energy sector. This confirms a result found in Myint and El-Halwagi [82] and Dunis et al. [44]. Still, Biodiesel production in the U.S is based predominantly on the use of Soybean Oil, precisely for the 82% [2].

In Table 9 the connection rate between the three considered sectors is shown. We divide the total number of connections that an entire sector has with another sector by the maximum number of possible connections between the two sectors considered. For example, there are 14 connections between metals and energy sectors, dividing by the maximum number of possible connections, i.e. 35, we get a ratio of 0.40. The highest connection rates lie on the main diagonal. Interestingly, the pandemic has strengthened the relationships between the commodities belonging to the same sector and weakened the inter-sectorial linkages. Before Covid-19, agriculture and energy commodities exhibited strong dependence also supported by other studies [13, 94, 104]. Such a link can be explained as a cause of the introduction of biofuels, that have intensified the ties between the sectors, as well as the need for efficient transport for perishable commodities like the agricultural ones. The degree of connectedness between the energy and agricultural commodity market, and between agricultural and metals, highlights the great effect produced by the financialization of commodity markets. The outbreak of Covid-19 has remarkably diminished the strength of inter-sectorial relationships. One hypothesis for this lies on the fact that during normal times trades are more liberalized while during periods of market turmoils the level of trust of investors is impaired. Moreover, in the specific event of the Covid-19 pandemic, the demand and supply economic chain has majorly hit with the abrupt interruptions of most economic activity therefore concerning the classes of commodities that are employed in the related industrial processes. Furthermore, we sort the considered commodities in terms of eigenvector centrality identifying the group of the "most connected" commodities.

Table 2 shows the eigenvector centrality scores. Soybean Oil is the most important in terms of centrality. This is explained by the fact that Soybean forms a large proportion (over 1/5-th) of the agricultural output of US farmers [2, 44], mainly because it is the most used agricultural commodity for biodiesel production in the US [82]. Cotton, Soybean Oil, and Copper are used as raw material or inputs for industries [12] and their prices are subject to demand-side shocks that are highly correlated, which can explain their prominent position in the eigenvector centrality. On the contrary, it is worth noting that Gold and Natural Gas UK lies in the lowest positions in terms of centrality. Less central nodes suggest a more stable and isolated behavior, highlighting safe-haven assets. As Balli et al. [12] point out in their study of commodity connectedness, Gold is known for his hedging abilities in crisis time and is therefore an alternative investment vehicle. We strengthen their findings ranking the position of commodities in the network with the use of eigenvector centrality and find that indeed that this commodity occupies one of the lowest positions in the Table 2.

**Fig. 2:**
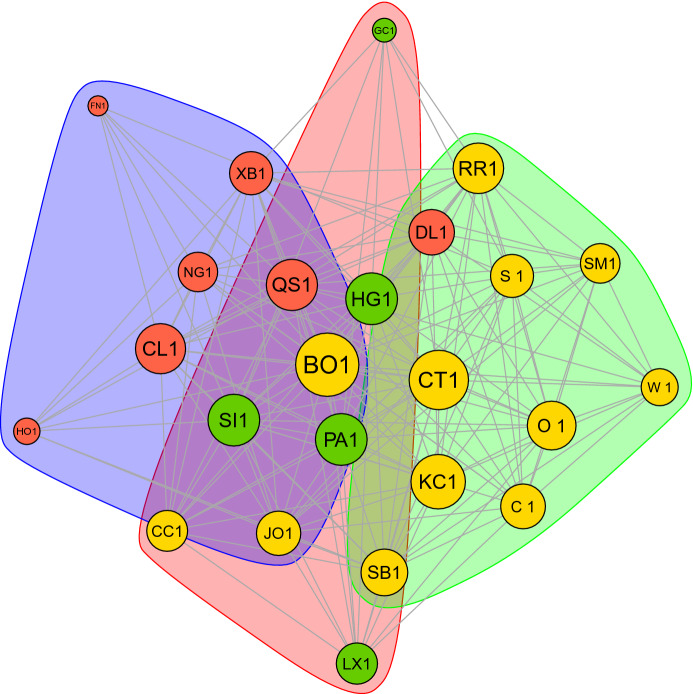
Community structure obtained via optimizing the modularity score. Size of the vertices change accordingly to the corresponding eigenvector centrality score. Sample period: Ocotber 3, 2005–March 25, 2022

Finally, to investigate the existence of clusters in the *network of commodity risk* we split the estimated graph into different clusters. We estimate three sub-graphs as presented in Fig. 2 and Table 10, which highlight the cluster for each commodity. The first cluster contains 6 nodes: it includes the entire Metal sector and one agricultural commodity (Cocoa). The second cluster contains 11 nodes: the entire agriculture sector, apart from Cocoa and Orange Juice, and Ethanol. The results confirm the strong interactions among Ethanol and agricultural commodities, which are generally higher than the interactions between oil and gas and agricultural markets [29]. The third cluster includes all the Energy commodities, apart from Ethanol, and Orange Juice. The results confirm the tendency of each node to attach preferably to a node of the same sector, with few exceptions.

**Table 2:** Eigenvector centrality scores of the commodities in the sample in decreasing order

	Eigenvector centrality
Soybean Oil	1.00
Cotton	0.94
Coffee	0.86
Copper	0.82
Silver	0.82
Palladium	0.82
Low Sulfur Gasolio	0.81
Rough Rice	0.80
WTI Crude Oil	0.79
Oats	0.77
Sugar	0.74
Ethanol	0.72
Corn	0.71
Orange Juice	0.70
Gasoline	0.68
Soybeans	0.68
Zinc	0.65
Cocoa	0.65
Soybean Meal	0.63
Natural Gas	0.62
Wheat	0.59
Heating Oil	0.42
Gold	0.38
Natural Gas UK	0.32

Sample period: October 3, 2005–March 25, 2022

## Conclusions

In this paper, we investigate the connectedness within commodity markets relying on a risk management perspective. Building upon the Sparse Graphical Lasso (GLASSO) methodology, we build the network of commodity risk basing on the residuals of GARCH-type models with underlying distribution selected for each commodity through a risk management approach. The risk management approach enters the model selection criteria designed to identify the best model for the assessment of the contribution of the commodities to the market risk. Such criteria exploits traditional backtesting procedures to evaluate the Value-at-Risk predictability of the set of the models considered. We apply the methodology to the sample of twenty-four commodity futures prices over the period that spans from October 3, 2005 to March 25, 2022.

Overall, we find that commodities show a moderate degree of connectedness within their network structure. The Covid-19 crisis has affected the interconnections increasing the heterogeneity within commodity markets as captured by the creation of three underlying clusters instead of the two that are estimated without including the pandemic in the sample period. However, the clusters identified mostly coincide with the three commodity sectors indicating that the additional heterogeneity is reflected in emphasized connections between commodities that belong to the same commodity sector. From the quantitative viewpoint, this finding is also endorsed by the lower eigenvector centrality scores, weakened degree of connections between the commodities and the other sectors, and density of the graph which has decreased by 18.6%. The pandemic has also influenced the proportion of optimal MS-GARCH models in the sample which has gone from 75% to 58%. The response of *the network of the commodity risks* to the recent persistent market uncertainty proves that global increasing connectedness does not represent a crisis stylized fact.

Our work is especially valuable to risk managers and policy-makers involved in the monitoring of the propagation of the commodity risk. Unlike previous studies on the assessment of the network of commodities, our methodology provides linkages that are determined conditionally on the ability of the models to forecast the VaR. In this way, the architecture of commodity markets depicts connections that incorporate information on the contribution to the market risk besides the underlying relationships that feature the commodities. This allows regulators and risk managers to infer more appropriate contribution of the commodity risks to the market risks since the risk exposure of each commodity has been conveniently modelled. Our findings reveal useful in this context providing information on the most relevant threats to the stability of commodity markets and, therefore, to design of tailored strategies for the mitigation of the commodity risk.

An immediate extension of our framework would consider either the application of the Gaussian Graphical model set-up to different network specifications or the implementation of non-Gaussian copulas. Another departure from our model is the consideration of long memory among the typical stylized facts of the volatility process. A third possible insight regards the employment of a more flexible class of models for the MS-GARCH, as the semi-Markov model where the sojourn-distribution is explicitly modelled rather than implicitly assumed to be geometric. This is the object of ongoing research.

## Figures and Tables

**Fig. 3:**
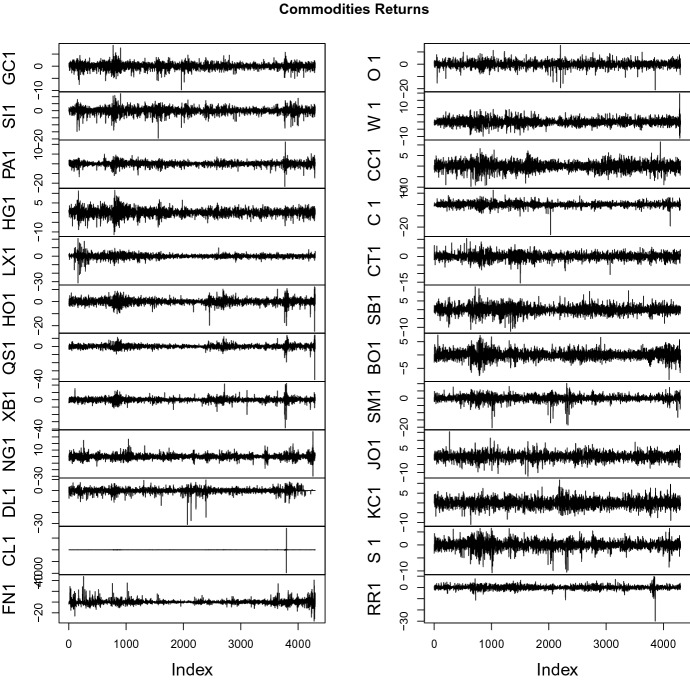
Log-returns of the commodities by sector. Agriculture: Coffee (KC1), Oats (O1), Soybeans (S1), Wheat (W1), Cocoa (CC1), Corn (C1), Rough Rice (RR1), Cotton (CT1), Sugar (SB1), Soybean Oil (BO1), Soybean Meal (SM1), Orange Juice (JO1). Energy: Gasoline (XB1), Heating Oil (HO1), Low Sulfur Gasolio (QS1), Natural Gas (NG1), Ethanol (DL1), WTI Crude Oil (CL1), Natural Gas UK (FN1). Precious Metals: Gold (GC1), Silver (SI1), Palladium (PA1). Industrial Metals: Copper (HG1), and Zinc (LX1). Sample period: October 3, 2005–March 25, 2022

## The appendix of the network of commodity risk

### Models specifications

#### GARCH models

Consider the time series of the log-return $y_t$ whereby $t=1,\dots , T$. According to the GARCH(1,1) model, $y_t$ can be decomposed into:

4 $$\begin{aligned} y_{t}=\mu +\epsilon _{t}, \end{aligned}$$

where the series of the residuals $\epsilon _t$ presents conditional heteroskedasticity. More specifically, $\epsilon _t=\sqrt{h_t}\;z_t$ and the innovation term $z_t$ follows the continuous standardized distribution $D_{\Theta }\left( 0,1,\right)$ with $\Theta$ as the set of parameters. The dynamics assumed for the conditional volatility $h_t$ is:

5 $$\begin{aligned} h_{t} = \alpha _{0}+\alpha _{1}\epsilon _{t-1}^2+\alpha _2 h_{t-1}, \end{aligned}$$

where $\alpha _0$, $\alpha _1$, and $\alpha _2$ are simultaneously estimated through maximization of the log-likelihood. To ensure the positiveness of $h_t$ and the covariance-stationarity, the parameters must satisfy respectively the conditions $\alpha _{0} > 0$, $\alpha _{1} \ge 0$, and $\alpha _2 \ge 0$ and $\alpha _1 + \alpha _2 < 1$.

#### MS-GARCH models

Unlike GARCH models, Markov-switching GARCH (MS-GARCH) models allow the coefficients $(\alpha _0,\alpha _1,\alpha _2, \Theta )$ to change over the regimes considered. According to the MS-GARCH(1,1) specification, the dynamics log-returns and the conditional volatility are described as follows:

6 $$\begin{aligned} y_{t}=\mu +\epsilon _{t}, \qquad \epsilon _{t}=\sqrt{h_{k_t}}\;\eta _{k_t}, \qquad \eta _{k_t} \vert (s_t=k,{\mathcal {F}}_{t-1}) \sim {\mathcal {D}}_{\Theta _k}(0,1), \end{aligned}$$

7 $$\begin{aligned} h_{k_t} = \alpha _{k_0}+\alpha _{k_1}\epsilon _{t-1}^2+\alpha _{k_2} h_{k_{t-1}}, \end{aligned}$$

where the latent variable $s_t$, defined on the discrete space $\{1,...,K\}$, evolves according to an unobserved first-order ergodic homogeneous Markov chain with transition probability matrix:

8 $$\begin{aligned} P= \begin{bmatrix} p_{1,1} &{} \cdots &{} p_{K,1}\\ \vdots &{} \ddots &{}\vdots \\ p_{1,K} &{} \cdots &{} p_{K,K} \end{bmatrix} \quad \text{ with } \quad p_{i,j}={\mathbb {P}}(s_t=j \vert s_{t-1}=i). \end{aligned}$$

For each regime, the estimation of the parameters of the model is carried out with Maximum Likelihood. As for the GARCH model, the positiveness of $h_{k_t}$ is obtained requires $\alpha _{k_0} > 0$, $\alpha _{k_1}> 0$, and $\alpha _{k_2} \ge 0$, while covariance-stationarity relies on $\alpha _{k_1}+\alpha _{k_2} < 1$, ($k=1,\dots ,K$).

#### Description of backtests

The Unconditional Coverage (UC) test of Kupiec [75] assesses the statistical significance of the frequency of exceedances of the log-returns over the VaR. The test builds upon the following system of hypothesis:

9 $$\begin{aligned} \left\{ \begin{matrix} H_0 : \pi = \tau \\ H_1 : \pi \ne \tau \end{matrix}\right. , \end{aligned}$$

with test statistics the likelihood ratio:

10 $$\begin{aligned} LR_{uc}(\tau ) = -2 \times \log \left[ \frac{L(\tau )}{L(\pi )}\right] = - 2 \times \log \left[ \frac{\tau ^{n_1} (1-\tau )^{T-n_1}}{\pi ^{n_1} (1-\pi )^{T-n_1}} \right] , \end{aligned}$$

where $n_1$ is the number of exceedances encountered. The statistic LRuc is asymptotically distributed as a chi-squared with one degree of freedom, $\chi ^2_1$.

The Conditional Coverage (CC) test of Christoffersen [32] gauges the time dependence between VaR exceedances. A first-order Markov structure for the dependence of the hits is given by the transition probability matrix $\Pi$:

11 $$\begin{aligned} \Pi = \left[ \begin{matrix} 1- \pi _{01} &{} \pi _{01} \\ 1- \pi _{11} &{} \pi _{11} \\ \end{matrix}\right] , \end{aligned}$$

with $\pi _{11}$ ($\pi _{10}$) that indicates the probability of observing a VaR exception in *t* given that in $t-1$ no violation is registered:

12 $$\begin{aligned} \begin{aligned} \pi _{11}&= {\mathbb {P}}(I_{t} = 1 | I_{t-1} = 1) \\ \pi _{10}&= {\mathbb {P}}(I_{t} = 1 | I_{t-1} = 0) \\ \end{aligned} \end{aligned}$$

and $1- \pi _{01}$ and $1- \pi _{11}$ are the respective probability of a non-violation in *t*. The independence hypothesis that $\pi _{01}=\pi _{11}$ is tested with the likelihood ratio:

13 $$\begin{aligned} LR_{cc} = -2 \log \frac{(1-\pi )^{n_0} \pi ^{n_1}}{(1-\pi _{01})^{n_{00}} \pi _{01}^{n_{01}} \pi _{11}^{n_{11}} (1-\pi _{11})^{n_{10}}}\sim \chi ^2_2 , \end{aligned}$$

where $n_0$ and $n_{00}$ are the number of times a non-violation is encountered and the number of times it is followed by another non-violation, respectively. On the contrary, $n_{11}$ is the number of times a violation is followed by another one, $n_{10}$ ($n_{01}$) counts the times a violation is followed by a non-violation (viceversa).

The Dynamic Quantile (DQ) test of Engle and Manganelli [45] can be interpreted as an overall goodness-of-fit test for the estimated VaR. Engle and Manganelli [45] consider that the conditional expectation of a quantile violation given any information known at $t-1$ should be exactly $\tau$. Hence, a linear regression model is set up where the sequence of violations represents the dependent variable while past violations or any other variables in the information set determine the set of explanatory variables. Denoting the set of parameters in the regression ${\hat{\delta }} =({\hat{\delta }}_0, ..., {\hat{\delta }}_{q-1})'$, and $\mathbf{Z }$ the corresponding data matrix with columns given by the observations for the *q* explanatory variables, the DQ test statistic for the null hypothesis of correct unconditional and conditional coverage is:

14 $$\begin{aligned} DQ_{1- \tau }=\frac{{\hat{\delta }}'\mathbf{Z }'\mathbf{Z }{\hat{\delta }}}{(1-\tau )\tau }. \ \ \end{aligned}$$

Under the null hypothesis, the test statistic is distributed as a $\chi ^2$ with *q* degrees of freedom where *q* is the number of lagged violations introduced in the aforementioned regression model.

### Figures

See Fig. 3.

### Tables

See Tables 3, 4, 5, 6, 7, 8, 9 and 10.

**Table 3:** Summary statistics of the daily log-returns of the commodities in the sample

	Mean	SD	Skewness	Kurtosis	J.B	L.B**	ARCH.LM	ADF
Gold	0.03	1.14	− 0.35	5.75	0.00	0.00	0.00	0.01
Silver	0.03	2.08	− 0.88	7.10	0.00	0.00	0.00	0.01
Palladium	0.06	2.12	− 0.55	10.49	0.00	0.00	0.00	0.01
Copper	0.02	1.74	− 0.14	4.07	0.00	0.00	0.00	0.01
Zinc	0.03	2.19	− 0.94	21.27	0.00	0.00	0.00	0.01
WTI Crude Oil	0.01	20.70	− 4.29	2084.02	0.00	0.00	0.00	0.01
Heating Oil	0.02	2.15	− 0.80	11.35	0.00	0.00	0.00	0.01
Low Sulfur Gasolio	0.01	2.11	− 1.64	38.33	0.00	0.00	0.00	0.01
Natural Gas	− 0.02	3.31	0.62	9.64	0.00	0.00	0.00	0.01
Gasoline	0.01	2.65	− 1.18	23.14	0.00	0.00	0.00	0.01
Natural Gas UK	0.04	3.99	1.58	21.08	0.00	0.00	0.00	0.01
Ethanol	− 0.00	2.03	− 2.44	29.37	0.00	0.00	0.00	0.01
Corn	0.03	1.87	− 0.93	14.46	0.00	0.00	0.00	0.01
Oats	0.03	2.19	− 0.59	7.93	0.00	0.00	0.00	0.01
Rough Rice	0.02	1.56	− 1.78	35.34	0.00	0.00	0.00	0.01
Soybeans	0.03	1.50	− 0.73	5.07	0.00	0.00	0.00	0.01
Wheat	0.03	2.07	0.24	3.88	0.00	0.00	0.00	0.01
Cocoa	0.01	1.79	− 0.21	2.42	0.00	0.00	0.00	0.01
Cotton	0.02	1.75	− 0.27	4.48	0.00	0.00	0.00	0.01
Coffee	0.02	1.96	0.14	1.96	0.00	0.00	0.00	0.01
Sugar	0.01	2.07	− 0.04	3.15	0.00	0.00	0.00	0.01
Soybean Oil	0.03	1.47	− 0.06	2.72	0.00	0.00	0.00	0.01
Soybean Meal	0.03	1.84	− 1.45	13.99	0.00	0.00	0.00	0.01
Orange Juice	0.01	2.06	− 0.01	2.98	0.00	0.00	0.00	0.01

We report the mean (Mean), standard deviation (SD), skewness, kurtosis, test statistic of the Jarque–Bera Test (J.B), test statistic of the Ljung–Box Test on the squared log-returns with 20 lags (L.B), test statistic of the ARCH Lagrange Multiplier Test (ARCH.LM), and test statistic of the Augmented Dickey–Fuller unit root test (ADF). We denote with c the significance at the 1% level. Sample period: October 3, 2005–March 25, 2022

** *p* values higher than the 1% significance level

**Table 4:** Summary of the works of reference on the literature on commodity markets

Study references	Study period	Methods	Commodity class	Summary
Fong and See [51]	1992–1997	GARCH, MSGARCH	Crude oil	The regime switching model performs noticeably better than non-switching models regardless of evaluation criteria
Cashin and McDermott [23]	1862–1999	Null Hypothesis to test	Industrial commodities	Commodities have shifted from a boom phase to a slump phase if prices have declined from their previous peak
Giot and Laurent [59]	1987–2002	RiskMetrics, Student APARCH and ARCH model	Metal, energy and agriculture commodities	Assess the performance of the RiskMetrics, skewed Student APARCH and skewed student ARCH models
Fong and See [52]	1992–1997	MSGARCH	Crude oil	Incorporating regime shifts improves the accuracy of short-term volatility forecasts
Baffes [10]	1960–2005	OLS regression	Primary Commodities	Effect of crude oil prices on the prices of 35 internationally traded primary commodities
Marimoutou et al. [78]	1983–2007	EVT models, GARCH, Historical Simulation and Filtered Historical	Crude oil	Extreme Value Theory and Filtered Historical Simulation procedures offer a major improvement over the traditional methods
Aloui and Mabrouk [5]	1987–2007	GARCH-type models	Energy commodities	Considering for long-range memory, fat-tails and asymmetry performs better in predicting a one- day-ahead VaR for both short and long trading positions
Tyner [104]	2006–2008	Price analysis	Energy and agriculture commodities	Exploration of the drivers in these markets as well as other major issues facing the corn ethanol industry in the United States such as the blend wall
Du et al. [43]	1998–2009	Stochastic volatility models	Oil and agriculture commodities	Linkage between crude oil volatility and agricultural commodity markets
Nazlioglu and Soytas [86]	1994–2010	Toda-Yamamoto; Nonparametric causality	Oil, agriculture and exchange rates	Nonlinear feedback relationship between the oil and the agricultural prices
Ewing and Malik [47]	1993–2010	GARCH	Gold and oil	Significant volatility transmission between gold and oil
Nazlioglu et al. [85]	1986–2011	Variance causality test	Oil and agriculture commodities	A shock to oil price volatility is transmitted to agricultural markets only in the post-crisis period
Spierdijk and Umar [99]	1970–2011	VAR	Agriculture, energy, industrial metals, live cattle, and precious metals commodities	Significant hedging ability for commodity futures indices
Smales [98]	2003–2012	Sentiment Analysis	Gold	Constraints imposed on traders have a significant impact on the net positions of both speculators and hedgers; this influences the way in which prices in the gold futures market react to news sentiment
Andriosopoulos and Nomikos [6]	2007–2010	Genetic and Differential Evolution algorithms	Energy and equity	The proposed methodology suggests an effective, and at the same time, least-expensive way to operate such a fund, giving the full flexibility of any investment style, long or short, that equities can provide
Reboredo [92]	2005–2013	Copulas	Oil and energy commodities	Oil and renewable energy displayed time-varying average and symmetric tail dependence
Rezitis [94]	1983–2013	VAR; Granger Causality	Agricultural commodity prices, crude oil prices and US dollar exchange rates	Bidirectional panel causality effects between crude oil prices and international agricultural prices as well as between US exchange rates and international agricultural prices
Joëts [70]	2005–2010	HAM	Energy commodities	The recent surge in energy prices is viewed as the consequence of irrational exuberance
de Nicola et al. [39]	1970–2013	VAR	Energy, agricultural and food commodities	Price returns of energy and agricultural commodities are highly correlated
Ji and Fan [69]	2000–2011	Graph theory	Crude oil	The integration of the world crude oil market is verified. Furthermore, the world crude oil market is characterised as a geographical and organisational structure
Barbaglia et al. [13]	2013–2015	Multi-class and VAR model; network analysis	Global, energy, metal and agricultural commodities	More common commodity price effects among portfolios than among markets
Kang et al. [72]	2002–2016	DECO-GARCH	Oil and agricultural commodities and precious metals	Strong spillover during crisis; Gold and silver are transmitters to other commodities
Algieri and Leccadito [4]	2005–2013	delta CoVaR	Energy, food and metals commodities	Commodity markets generate contagion risks which are mainly triggered by financial factors for energy and metal markets and by financial and economic fundamentals for food markets
Mensi et al. [81]	1998–2016	VMD method and static and time-varying symmetric and asymmetric copula functions	Commodities and equity	The dependence structure varies across market conditions and under investment horizons; risk spillovers are higher in the long than the short run investment horizon
Diebold et al. [41]	2011–2016	VAR; FEVD; Network Analysis	Energy, livestock and agricultural commodities, precious and industrial metals	Clustering of commodities into groups; high overall connectedness and energy sector sends shocks to other commodities
Rehman et al. [93]	1989–2016	SVAR	Crude oil, precious and industrial metals	Structural oil shocks impact precious metal returns tails except gold
Ferrer et al. [49]	2003–2017	VAR; FEVD	Crude oil, US renewable energy stocks, high technology stocks, conventional energy stocks, US 10-year Treasury bond yields	Most of return and volatility connectedness is found in the short-term; Crude oil prices are not the key driver of renewable energy companies’ performance
Laporta et al. [76]	2002–2017	GARCH, GAS and CAViaR models; Dynamic Quantile Regression	Energy commodities	CAViaR and DQR models provide more accurate VaR estimates at high confidence levels
Ji et al. [68]	2000–2017	CoVaR, delta CoVaR, dependence-switching copula	Energy and agricultural commodities	Lower tail dependence is stronger in bearish regime than in bullish one; agricultural commodities are more sensitive to shock from oil than from gas
Shen et al. [97]	2000–2014	VAR	Energy commodities	Asymmetric patterns in response of gains and losses transmission between energy markets; Extreme market risk is more easily transmitted across markets than moderate risk
Zhang and Broadstock [115]	1982–2017	VAR; FEVD; Granger Causality; Network Analysis	Beverage, Fertilizers, Food, Metal, Precious metal, Raw materials, Oil	Significant rising of connectedness has been found after the global financial crisis
Tiwari et al. [102]	2007–2013	Wavelet analysis	Oil and energy commodities	During the shale gas revolution period of 2007-2013, oil and natural gas prices were procyclical and oil prices were leading natural gas prices
Balli et al. [12]	2007–2016	GDFM; SV models	Energy commodities, precious and industrial metals, and agricultural commodities	Spillovers increase during the GFC and 2014-16 oil price collapse; Network analysis shows more spillover within a specific commodity class
Ramiah et al. [91]	1990–2017	Non-parametric ranking test and kernel regression	Metal, chemical, precious, energy and agriculture commodities	There is a delayed reaction of investor in commodity markets compared to the equity market
Malik and Umar [77]	1996–2019	VAR	Exchange rates of major oil-exporting and oil-importing countries	Results show that demand shocks have a major impact while supply shocks have no impact
Zaremba et al. [112]	1265–2017	Wavelet analysis	Agriculture, energy, industrial commodities	Robust inflation hedging properties of agricultural, energy, and industrial commodities for the 4- to 8-year horizon through almost the entire seven centuries
Nguyen et al. [88]	1992–2017	local Gaussian correlation measure	Commodity and U.S. financial markets	Financialization hypothesis is confirmed; Special role of Gold is highlighted
Christodoulakis [30]	2001–2013	GMM	Energy, agriculture, livestock, industrial metals and precious metals	Joint preference asymmetries for longer maturities, joint preference symmetries for short maturities
Naeem et al. [83]	2007–2018	EGARCH-Copula approach	Energy and commodity ETFs, oil	Positive correlations of energy and commodity ETFs with oil prices is found
Flori et al. [50]	1980–2017	Graph-theoretical approach; Granger causal connectivity analysis	Commodities and climate related variables	Climate conditions affect financial stability by impacting commodity comovements
Umar et al. [105]	2020–2020	Wavelet coherence and phase-difference methods	Energy commodities, precious and agricultural commodities	Non-precious metals offer the best diversification during the recovery from crisis
Zaremba et al. [113]	1850–2019	Pearson’s product-moment pairwise correlation coefficients; Gerber statistic; $R^2$ analysis.	Precious metals, energy, industrials, and agriculturals	Findings cast doubt on the link between commodity dependence and financialisation
Balcilar et al. [11]	1990–2019	TVP-VAR	Crude Oil and agricultural commodities	Crude Oil not only affects commodity assets but is also equally responsive to their innovations
Tiwari et al. [103]	1986–2018	time-varying generalised Hurst exponent	Energy commodities	After the subprime crisis, the persistence of energy spot market products has increased
Umar et al. [106]	2000–2020	TVP-VAR	Oil and agriculture commodities	Higher directional return and volatility connectedness to oil risk and demand than supply shocks
Umar et al. [107]	2020–2021	TVP-VAR	Agricultural and livestock commodity, Coronavirus Media Coverage Index	Dynamic total return and volatility connectedness fluctuate over time, reaching a peak during both the first and the third waves of the global pandemic crisis

We report study reference, the study period, core method employed in the analysis (Methods), the commodity sectors on which the analysis is focused (Commodity class), and a brief description of the major findings (Summary)

**Table 5:** Backtesting results for the commodities Gold, Palladium, Silver, Zinc, and Copper (Metals commodity sector)

Confidence level	95%				99%			
	uc.LRp	cc.LRp	DQp	AE	uc.LRp	cc.LRp	DQp	AE
*Gold*								
GARCHnorm	0.376169**	0.673278**	0.272021**	0.926612	1.8e−05	7e−06	0	1.927354
MSGARCHnorm	0.538857**	0.768622**	0.318919**	0.948851	0.848702**	0.078492**	0.025813	0.963677
GARCHstd	0.377801**	0.646796**	0.514667**	1.07487	0.996913**	0.09181**	0.075842**	1.000741
MSGARCHstd	0.992953**	0.952407**	0.353444**	1.000741	0.191705**	0.013278	0.000506	1.260193
GARCHged	0.481144**	0.780227**	0.547514**	0.941438	0.844493**	0.010177	4.4e−05	1.037806
MSGARCHged	0.866417**	0.959651**	0.775744**	0.985915	0.447366**	0.001146	0	1.148999
GARCHsnorm	0.247085**	0.500426**	0.212008**	0.904374	0.000457	7.3e−05	0	1.742031
MSGARCHsnorm	0.046827	0.116861**	0.051335**	0.837658	0.848702**	0.078492**	0.016064	0.963677
GARCHsstd	0.784965**	0.87801**	0.525519**	1.02298	0.69809**	0.064088**	0.035234	0.926612
MSGARCHsstd	0.729269**	0.792302**	0.250049**	0.97109	0.844493**	0.010177	4.7e−05	1.037806
GARCHsged	0.329276**	0.616101**	0.407365**	0.919199	0.69809**	0.064088**	0.046315	0.926612
MSGARCHsged	0.599692**	0.869062**	0.142255**	0.956264	0.844493**	0.010177	3.3e−05	1.037806
*Palladium*								
GARCHnorm	0.922692**	0.038134	0.055372**	1.008154	0	0	0	2.149741
MSGARCHnorm	0.220265**	0.001177	0.000205	1.104522	0.004327	0.00181	1.8e−05**	1.593773
GARCHstd	0.220265**	0.036004	0.012632	1.104522	0.011746	0.003498	0.000101	1.519644
MSGARCHstd	0.53389**	0.012154	0.007221	1.052632	0.011746	0.003498	0.000163	1.519644
GARCHged	0.922692**	0.038134	0.053203**	1.008154	0.007206	0.002552	7.5e−05**	1.556709
MSGARCHged	0.718505**	0.022332	0.009323	1.030393	0.000457	0.000348	0	1.742031
GARCHsnorm	0.329276**	0.004679	0.013865	0.919199	9e−06	4e−06	0	1.964418
MSGARCHsnorm	0.599692**	0.033631	0.016613	0.956264	0.004327	0.00181	4.3e−05	1.593773
GARCHsstd	0.797141**	0.022501	0.028856	0.978503	0.137853**	0.012129	0.000299	1.297257
MSGARCHsstd	0.6633**	0.087649**	0.045702	0.963677	0.260377**	0.014018	0.000167	1.223128
GARCHsged	0.329276**	0.004679	0.01127	0.919199	0.029222	0.00601	0.00022	1.445515
MSGARCHsged	0.426842**	0.046269	0.062604**	0.934025	0.096832**	0.010698	0.000293	1.334322
*Silver*								
GARCHnorm	0.426842**	0.728406**	0.01555	0.934025	0	0	0	2.149741
MSGARCHnorm	0.93306**	0.644346**	0.090229**	0.99296	0.066456**	0.049959	0	1.371386
GARCHstd	0.163877**	0.370471**	0.082125**	1.118933	0.191705**	0.013278	1e−04**	1.260193
MSGARCHstd	0.536826**	0.815249**	0.096226**	1.052242	0.004327	0.00181	0	1.593773
GARCHged	0.481144**	0.712248**	0.067553**	0.941438	0.191705**	0.013278	8.6e−05**	1.260193
MSGARCHged	0.797141**	0.944272**	0.042973	0.978503	0.011746	0.003498	0	1.519644
GARCHsnorm	0.180153**	0.390089**	0.017609	0.889548	4e−06**	0	0	2.001483
MSGARCHsnorm	0.284285**	0.46621**	0.037643	0.911449	0.699371**	0.11023**	0	1.07487
GARCHsstd	0.428915**	0.642174**	0.088059**	1.067062	0.699371**	0.0118	1.2e−05**	1.07487
MSGARCHsstd	0.793748**	0.574531**	0.038624	0.97814	0.565949**	0.113531**	9e−06**	1.111935
GARCHsged	0.376169**	0.593415**	0.02916	0.926612	0.447366**	0.013942	2.9e−05**	1.148999
MSGARCHsged	0.535971**	0.759965**	0.045742	0.948499	0.844493**	0.102753**	0.000173	1.037806
*Zinc*								
GARCHnorm	0.936567**	0.440549**	0.128704**	0.993328	0.260377**	0.384782**	0.35774**	1.223128
MSGARCHnorm	0.922692**	0.489923**	0.001407	1.008154	0.844493**	0.569028**	0.1717**	1.037806
GARCHstd	0.85319**	0.509112**	0.010503	1.015567	0.556782**	0.384266**	0.397618**	0.889548
MSGARCHstd	0.85319**	0.723387**	0.02322	1.015567	0.429471**	0.311619**	0.290963**	0.852483
GARCHged	0.797141**	0.380745**	0.020441	0.978503	0.429471**	0.311619**	0.343904**	0.852483
MSGARCHged	0.797141**	0.588467**	0.033023	0.978503	0.556782**	0.384266**	0.175018**	0.889548
GARCHsnorm	0.797141**	0.380745**	0.083603**	0.978503	0.447366**	0.501277**	0.5536**	1.148999
MSGARCHsnorm	0.718505**	0.889211**	0.037931	1.030393	0.699371**	0.566416**	0.240576**	1.07487
GARCHsstd	0.936567**	0.440549**	0.020503	0.993328	0.429471**	0.60052**	0.670183**	0.852483
MSGARCHsstd	0.784965**	0.523944**	0.068473**	1.02298	0.319515**	0.240722**	0.58721**	0.815419
GARCHsged	0.729269**	0.348669**	0.039292	0.97109	0.228641**	0.410883**	0.419957**	0.778354
MSGARCHsged	0.797141**	0.800231**	0.05046**	0.978503	0.556782**	0.384266**	0.294271**	0.889548
*Copper*								
GARCHnorm	0.247085**	0.417994**	0.479086**	0.904374	0.000826	0.000109	0	1.704967
MSGARCHnorm	0.599692**	0.869062**	0.837004**	0.956264	0.018733	0.000678	0	1.48258
GARCHstd	0.866417**	0.836154**	0.547015**	0.985915	0.260377**	0.001327	0	1.223128
MSGARCHstd	0.481144**	0.780227**	0.855243**	0.941438	0.096832**	0.001232	0	1.334322
GARCHged	0.286243**	0.47466**	0.539922**	0.911787	0.447366**	0.001146	0	1.148999
MSGARCHged	0.329276**	0.616101**	0.058863**	0.919199	0.191705**	0.001345	0	1.260193
GARCHsnorm	0.211755**	0.364273**	0.417435**	0.896961	0.004327	0.00031	0	1.593773
MSGARCHsnorm	0.286243**	0.558099**	0.68568**	0.911787	0.066456**	0.001117	0	1.371386
GARCHsstd	0.6633**	0.711657**	0.523842**	0.963677	0.345368**	0.001259	0	1.186064
MSGARCHsstd	0.329276**	0.533458**	0.617516**	0.919199	0.260377**	0.001327	0	1.223128
GARCHsged	0.286243**	0.47466**	0.526265**	0.911787	0.565949**	0.000999	0	1.111935
MSGARCHsged	0.180153**	0.390089**	0.406984**	0.889548	0.260377**	0.001327	0	1.223128

We report the *p* values of the Unconditional Coverage (uc.LRp), Conditional Coverage (cc.LRp), Dynamic Quantile (DQp) tests and the Actual over Expected Exceedances Ratio (AE) computed at the 95% and 99% confidence levels. The models included in the assessment are GARCH(1,1) with innovations following a Normal (GARCHnorm), Skewed Normal (GARCHsnorm), Student’s-t (GARCHstd), Skewed Student’s-t (GARCHsstd), Generalized Error Distribution (GARCHged), and Skewed Generalized Error Distribution (GARCHsged) and MS-GARCH(1,1) following a Normal (MSGARCHnorm), Skewed Normal (MSGARCHsnorm), Student’s-t (MSGARCHstd), Skewed Student’s-t (MSGARCHsstd), Generalized Error Distribution (MSGARCHged), and Skewed Generalized Error Distribution (MSGARCHsged)

* Denotes the *p* values higher than the 5% significance level

** Denotes the *p* values higher than the 1% significance level

Sample period: October 3, 2005–March 25, 2022

**Table 6:** Backtesting results for the commodities WTI, Natural Gas UK, Heating Oil, Ethanol, L.S Gasolio, Gasoline, Natural Gas (Energy commodity sector)

Confidence level	95%				99%			
	uc.LRp	cc.LRp	DQp	AE	uc.LRp	cc.LRp	DQp	AE
*WTI*								
GARCHnorm	0.286243**	0.215639**	0.324009**	0.911787	0.000457	0.002108	0.998099**	1.742031
MSGARCHnorm	0	0	0.999998**	9.870322	0	0	1**	49.351612
GARCHstd	0.162516**	0.324307**	0.993754**	1.119348	0.018733	0.05598**	0.999999**	1.48258
MSGARCHstd	0.138392**	0.063562**	0.99884**	1.126761	0.011746	0.037856	1**	1.519644
GARCHged	0.6633**	0.505691**	0.998082**	0.963677	0.096832**	0.201986**	1**	1.334322
MSGARCHged	0.426311**	0.50965**	0.987309**	1.067457	0.007206	0.024923	1**	1.556709
GARCHsnorm	0.07161**	0.079492**	0.769626**	0.852483	0.007206	0.024923	0.999884**	1.556709
MSGARCHsnorm	0	0	0.999998**	9.870322	0	0	1**	49.351612
GARCHsstd	0.922692**	0.707753**	0.989617**	1.008154	0.096832**	0.201986**	0.999997**	1.334322
MSGARCHsstd	0.85319**	0.179494**	0.994373**	1.015567	0.096832**	0.201986**	0.999999**	1.334322
GARCHsged	0.426842**	0.499202**	0.981284**	0.934025	0.345368**	0.446807**	0.999988**	1.186064
MSGARCHsged	0	0	0.988484**	1.452928	0	0	0.999999**	3.854707
*Natural Gas UK*								
GARCHnorm	0.481144**	0.009157	0.000199	0.941438	0.018733	0.021533	0	1.48258
MSGARCHnorm	0.038177	0.006409	2.00E−06	1.178651	0.565949**	0.113531**	0	1.111935
GARCHstd	0.031028	0.001312	1.8e−05	1.186064	0.447366**	0.501277**	0	1.148999
MSGARCHstd	0.162516**	0.007884	5.00E−06	1.119348	0.066456**	0.153107**	0	1.371386
GARCHged	0.538857**	0.011202	0.000345	0.948851	0.996913**	0.549507**	0	1.000741
MSGARCHged	0.162516**	0.007884	7.8e−05	1.119348	0.096832**	0.062199**	0	1.334322
GARCHsnorm	0.332944**	0.006588	0.000186	1.082283	0.000131	3.00E−05	0	1.81616
MSGARCHsnorm	0.003693	0.000623	0	1.25278	0.018733	0.004655	0	1.48258
GARCHsstd	0.000689	3.1e−05	0	1.297257	0.137853**	0.25846**	0	1.297257
MSGARCHsstd	0.01008	0.000949	0	1.223128	0.044571	0.038892	0	1.408451
GARCHsged	0.592603**	0.026594	0.00089	1.045219	0.191705**	0.3205**	0	1.260193
MSGARCHsged	0	0	0	10.455724	0	0	0	52.278622
*Heating Oil*								
GARCHnorm	0.53389**	0.693124**	0.120038**	1.052632	0.001464	0.006095	0.00027	1.667902
MSGARCHnorm	0.0467	0.137776**	0.007206	1.171238	0.029222	0.080541**	0.008966	1.445515
GARCHstd	0.068662**	0.178165**	0.020561	1.156412	0.191705**	0.3205**	0.010064	1.260193
MSGARCHstd	0.098622**	0.24531**	0.038368	1.141586	0.191705**	0.3205**	0.007833	1.260193
GARCHged	0.592603**	0.745351**	0.099405**	1.045219	0.447366**	0.501277**	0.029084	1.148999
MSGARCHged	0.098622**	0.233768**	0.022659	1.141586	0.007206	0.024923	0.001097	1.556709
GARCHsnorm	0.784965**	0.963175**	0.215632**	1.02298	0.007206	0.024923	0.00079	1.556709
MSGARCHsnorm	0.098622**	0.24531**	0.029798	1.141586	0.096832**	0.201986**	0.008677	1.334322
GARCHsstd	0.162516**	0.371858**	0.031443	1.119348	0.699371**	0.566416**	0.033559	1.07487
MSGARCHsstd	0.068662**	0.190635**	0.023278	1.156412	0.137853**	0.25846**	0.004244	1.297257
GARCHsged	0.654254**	0.794345**	0.091228**	1.037806	0.565949**	0.542742**	0.033717	1.111935
MSGARCHsged	0.332944**	0.624808**	0.056914**	1.082283	0.096832**	0.201986**	0.038381	1.334322
*Ethanol*								
GARCHnorm	0.001242	8.7e−05	0.003903	0.741015	0.018838	0.004379	7.4e−05	1.48203
MSGARCHnorm	0	0	0	11.70804	0	0	0	58.5402
GARCHstd	4.00E−06	0	0	1.407929	0	0	0	3.853279
MSGARCHstd	0	0	0	1.682104	0	0	0	3.001112
GARCHged	0.105096**	0	0	0.866988	0.007252	0	0	1.556132
MSGARCHged	0	0	0	11.70804	0	0	0	58.5402
GARCHsnorm	5.9e−05	4.4e−05	0.002908	0.681734	0.138392**	0.254925**	0.585762**	1.296777
MSGARCHsnorm	0.139598**	0	0.051353**	1.126343	4.00E−06	0	0.75966**	2.000741
GARCHsstd	7.7e−05	0	0	1.348648	0	0	0	3.705076
MSGARCHsstd	1.00E−06	0	0	1.44498	0	0	0.054491**	2.260096
GARCHsged	0.327124**	0	0	0.918859	0.000133	0	0	1.815487
MSGARCHsged	0.481164**	0	0.013414	1.059652	0	0	0.113669**	2.111893
*L.S.Gasolio*								
GARCHnorm	0.016083	0.008514	0.013422	1.208302	0	3.00E−06	0	2.112676
MSGARCHnorm	0.003693	0.003696	0.012349	1.25278	1.8e−05	6.6e−05	9.00E−06	1.927354
GARCHstd	0.000201	0.000119	9.8e−05	1.326909	0.002544	0.009995	0.021922	1.630838
MSGARCHstd	0.000924	0.000303	0.000321	1.289844	0.011746	0.01534	0.007292	1.519644
GARCHged	0.020139	0.009492	0.014018	1.20089	0.137853**	0.25846**	0.611293**	1.297257
MSGARCHged	0.02507	0.005551	0.004621	1.193477	0.011746	0.037856	0.248321**	1.519644
GARCHsnorm	0.02507	0.010489	0.014526	1.193477	2.00E−06	1.2e−05	1.00E−06	2.038547
MSGARCHsnorm	0.08253**	0.030287	0.023212	1.148999	0.000131	0.000391	3.9e−05	1.81616
GARCHsstd	0.001637	0.001343	0.001067	1.275019	0.044571	0.112663**	0.084314**	1.408451
MSGARCHsstd	0.020139	0.002545	0.002498	1.20089	0.066456**	0.049959	0.011816	1.371386
GARCHsged	0.038177	0.012473	0.012606	1.178651	0.066456**	0.153107**	0.097539**	1.371386
MSGARCHsged	0.138392**	0.107628**	0.026985	1.126761	0.260377**	0.384782**	0.689165**	1.223128
*Gasoline*								
GARCHnorm	0.797141**	0.949723**	0.999712**	0.978503	1.00E−06	5.00E−06	1.00E−06	2.075612
MSGARCHnorm	0.098622**	0.24531**	0.429401**	1.141586	0.096832**	0.201986**	0.067329**	1.334322
GARCHstd	0.332944**	0.589478**	0.655888**	1.082283	0.044571	0.112663**	0.040811	1.408451
MSGARCHstd	0.377801**	0.646796**	0.465694**	1.07487	0.011746	0.037856	0.035793	1.519644
GARCHged	0.797141**	0.944272**	0.835113**	0.978503	0.565949**	0.605186**	0.228264**	1.111935
MSGARCHged	0.291763**	0.532529**	0.730573**	1.089696	0.044571	0.112663**	0.052285**	1.408451
GARCHsnorm	0.426842**	0.67518**	0.977582**	0.934025	1.8e−05	6.6e−05	8.8e−05	1.927354
MSGARCHsnorm	0.25423**	0.495833**	0.614729**	1.097109	0.066456**	0.153107**	0.076079**	1.371386
GARCHsstd	0.53389**	0.809986**	0.677071**	1.052632	0.565949**	0.605186**	0.260247**	1.111935
MSGARCHsstd	0.377801**	0.646796**	0.121419**	1.07487	0.191705**	0.3205**	0.064282**	1.260193
GARCHsged	0.866417**	0.959651**	0.712483**	0.985915	0.260377**	0.352689**	0.18532**	1.223128
MSGARCHsged	0.729269**	0.758381**	0.914729**	0.97109	0.137853**	0.25846**	0.06866**	1.297257
*Natural Gas*								
GARCHnorm	0.106023**	0.248188**	0.000613	0.867309	0.260377**	0.352689**	0	1.223128
MSGARCHnorm	0.481144**	0.373426**	0.000525	0.941438	0.137853**	0.209911**	0	1.297257
GARCHstd	0.286243**	0.47466**	0.000333	0.911787	0.556782**	0.678314**	0	0.889548
MSGARCHstd	0.6633**	0.711657**	0.00055	0.963677	0.345368**	0.436289**	0	1.186064
GARCHged	0.046827	0.116861**	0.000287	0.837658	0.228641**	0.410883**	0	0.778354
MSGARCHged	0.211755**	0.254273**	0.000536	0.896961	0.844493**	0.73126**	0	1.037806
GARCHsnorm	0.599692**	0.821237**	0.001347	0.956264	0.011746	0.022208	0	1.519644
MSGARCHsnorm	0.797141**	0.800231**	0.000541	0.978503	0.011746	0.022208	0	1.519644
GARCHsstd	0.936567**	0.86523**	0.001241	0.993328	0.996913**	0.761058**	0	1.000741
MSGARCHsstd	0.592603**	0.841768**	0.00054	1.045219	0.018733	0.03455	0	1.48258
GARCHsged	0.211755**	0.444122**	0.00056	0.896961	0.996913**	0.761058**	0	1.000741
MSGARCHsged	0.599692**	0.661202**	0.001528	0.956264	0.066456**	0.110924**	0	1.371386

We report the *p* values of the Unconditional Coverage (uc.LRp), Conditional Coverage (cc.LRp), Dynamic Quantile (DQp) tests and the Actual over Expected Exceedances Ratio (AE) computed at the 95% and 99% confidence levels. The models included in the assessment are GARCH(1,1) with innovations following a Normal (GARCHnorm), Skewed Normal (GARCHsnorm), Student’s-t (GARCHstd), Skewed Student’s-t (GARCHsstd), Generalized Error Distribution (GARCHged), and Skewed Generalized Error Distribution (GARCHsged) and MS-GARCH(1,1) following a Normal (MSGARCHnorm), Skewed Normal (MSGARCHsnorm), Student’s-t (MSGARCHstd), Skewed Student’s-t (MSGARCHsstd), Generalized Error Distribution (MSGARCHged), and Skewed Generalized Error Distribution (MSGARCHsged)

* Denotes the *p* values higher than the 5% significance level

** Denotes the *p* values higher than the 1% significance level

Sample period: October 3, 2005–March 25, 2022

**Table 7:** Backtesting results for the commodities Corn, Cocoa, Oats, Cotton, Rough Rice, Coffee, Soybeans, Sugar, Wheat, Soybean Oil, Orange Juice, Soybean Meal (Agriculture commodity sector)

Confidence level	95%				99%			
	uc.LRp	cc.LRp	DQp	AE	uc.LRp	cc.LRp	DQp	AE
*Corn*								
GARCHnorm	1e−06**	0	9.3e−05**	0.615271	0.011746	6.1e−05**	0	1.519644
MSGARCHnorm	0.087482**	0.011279	0.036897	0.859896	0.191705**	0.013278	8.7e−05**	1.260193
GARCHstd	0.023255	0.000595	0.010703	0.815419	0.447366**	0.112431**	0.089284**	1.148999
MSGARCHstd	0.087482**	0.02633	0.094703**	0.859896	0.137853**	0.001312	0	1.297257
GARCHged	0	0	1.8e−05**	0.570793	0.102975**	0.231256**	0.29938**	0.704225
MSGARCHged	0.002433	0.000638	0.019307	0.756116	0.447366**	0.013942	0.000126	1.148999
GARCHsnorm	1e−06**	0	0.000183	0.607858	0.066456**	0.001117	1e−06**	1.371386
MSGARCHsnorm	0.037398	0.003423	0.032947	0.830245	0.565949**	0.013098	0.000222	1.111935
GARCHsstd	0.01068	0.000187	0.004173	0.79318	0.699371**	0.11023**	0.059708**	1.07487
MSGARCHsstd	0.013966	0.002355	0.014881	0.800593	0.345368**	0.001259	0	1.186064
GARCHsged	2e−06**	0	6e−05**	0.630096	0.996913**	0.09181**	0.034724	1.000741
MSGARCHsged	0.003333	0.00035	0.001891	0.763529	0.066456**	0.001117	0	1.371386
*Cocoa*								
GARCHnorm	0.481144**	0.373426**	0.404176**	0.941438	0.007206	0.000415	0	1.556709
MSGARCHnorm	0.729269**	0.197239**	0.133631**	0.97109	0.029222	0.02937	0.000264	1.445515
GARCHstd	0.922692**	0.489923**	0.531656**	1.008154	0.565949**	0.113531**	0.027145	1.111935
MSGARCHstd	0.936567**	0.262422**	0.16268**	0.993328	0.447366**	0.112431**	0.006144	1.148999
GARCHged	0.180153**	0.127769**	0.29398**	0.889548	0.848702**	0.509321**	0.455834**	0.963677
MSGARCHged	0.329276**	0.03151	0.009584	0.919199	0.699371**	0.566416**	0.040742	1.07487
GARCHsnorm	0.329276**	0.251301**	0.332859**	0.919199	0.018733	0.000678	0	1.48258
MSGARCHsnorm	0.538857**	0.251877**	0.083059**	0.948851	0.096832**	0.201986**	0.051822**	1.334322
GARCHsstd	0.729269**	0.348669**	0.355458**	0.97109	0.844493**	0.102753**	0.021506	1.037806
MSGARCHsstd	0.866417**	0.24103**	0.109761**	0.985915	0.699371**	0.566416**	0.423868**	1.07487
GARCHsged	0.247085**	0.183078**	0.396397**	0.904374	0.848702**	0.509321**	0.601433**	0.963677
MSGARCHsged	0.426842**	0.099668**	0.072971**	0.934025	0.69809**	0.452346**	0.554777**	0.926612
*Oats*								
GARCHnorm	0.010551	0.025755	0.028003	0.792886	0.191705**	0.3205**	0.45965**	1.260193
MSGARCHnorm	0.793748**	0.201993**	0.105016**	0.97814	0.319515**	0.240722**	0.702016**	0.815419
GARCHstd	0.856665**	0.483646**	0.083857**	1.015191	0.848702**	0.509321**	0.836011**	0.963677
MSGARCHstd	0.660119**	0.161073**	0.256858**	0.96332	0.844493**	0.569028**	0.633835**	1.037806
GARCHged	0.046355	0.078011**	0.031655	0.837347	0.228641**	0.176721**	0.594603**	0.778354
MSGARCHged	0.478429**	0.106085**	0.050126**	0.941089	0.996913**	0.549507**	0.877766**	1.000741
GARCHsnorm	0.007993	0.019337	0.026297	0.785476	0.191705**	0.3205**	0.459643**	1.260193
MSGARCHsnorm	0.793748**	0.201993**	0.097417**	0.97814	0.102975**	0.231256**	0.790905**	0.704225
GARCHsstd	0.856665**	0.483646**	0.110756**	1.015191	0.996913**	0.549507**	0.844961**	1.000741
MSGARCHsstd	0.862953**	0.115043**	0.012937	0.98555	0.565949**	0.113531**	9.8e−05**	1.111935
GARCHsged	0.070937**	0.121375**	0.046818	0.852167	0.429471**	0.311619**	0.763183**	0.852483
MSGARCHsged	0.327124**	0.129665**	0.037439	0.918859	0.848702**	0.509321**	0.662643**	0.963677
*Cotton*								
GARCHnorm	0.426842**	0.099668**	0.111656**	0.934025	0.000247	0.000216	1.4e−05	1.779096
MSGARCHnorm	0.784965**	0.098907**	0.170881**	1.02298	0.191705**	0.3205**	0.475666**	1.260193
GARCHstd	0.654254**	0.112376**	0.199126**	1.037806	0.345368**	0.446807**	0.610373**	1.186064
MSGARCHstd	0.936567**	0.067909**	0.120201**	0.993328	0.345368**	0.446807**	0.67507**	1.186064
GARCHged	0.329276**	0.141126**	0.200114**	0.919199	0.996913**	0.09181**	0.062845**	1.000741
MSGARCHged	0.211755**	0.038545	0.080121**	0.896961	0.345368**	0.107205**	0.007552	1.186064
GARCHsnorm	0.180153**	0.030838	0.050062**	0.889548	0.000826	0.000547	7.2e−05	1.704967
MSGARCHsnorm	0.866417**	0.126714**	0.055129**	0.985915	0.565949**	0.542742**	0.535499**	1.111935
GARCHsstd	0.718505**	0.105951**	0.137861**	1.030393	0.137853**	0.074986**	0.082556**	1.297257
MSGARCHsstd	0.797141**	0.05291**	0.049236	0.978503	0.191705**	0.087449**	0.06821**	1.260193
GARCHsged	0.329276**	0.141126**	0.187072**	0.919199	0.996913**	0.09181**	0.06281**	1.000741
MSGARCHsged	0.599692**	0.033631	0.058223**	0.956264	0.345368**	0.107205**	0.062089**	1.186064
*Rough.Rice*								
GARCHnorm	0.037398	0.000112	0.001064	0.830245	0.260377**	0.098552**	0.127786**	1.223128
MSGARCHnorm	0.106023**	0.000653	0.004699	0.867309	0.556782**	0.384266**	0.700468**	0.889548
GARCHstd	0.729269**	0.000836	0.000241	0.97109	0.429471**	0.036898	0.015663	0.852483
MSGARCHstd	0.426842**	6.7e−05	2.3e−05	0.934025	0.69809**	0.452346**	0.83786**	0.926612
GARCHged	0.013966	1.00E−06	7.00E−06	0.800593	0.429471**	0.036898	0.010444	0.852483
MSGARCHged	0	0	0	10.778354	0	0	0	53.891772
GARCHsnorm	0.058145**	0.000235	0.002011	0.84507	0.191705**	0.087449**	0.134392**	1.260193
MSGARCHsnorm	0.936567**	0.001529	0.000183	0.993328	0.447366**	0.501277**	0.666702**	1.148999
GARCHsstd	0.592603**	0.00154	0.001052	1.045219	0.996913**	0.008388	3.5e−05	1.000741
MSGARCHsstd	0.992953**	0.000591	3.7e−05	1.000741	0.137853**	0.012129	0.000517	1.297257
GARCHsged	0.247085**	0.000271	0.001173	0.904374	0.69809**	0.064088**	0.032217	0.926612
MSGARCHsged	0.481144**	0.001061	0.001119	0.941438	0.044571	0.000978	0	1.408451
*Coffee*								
GARCHnorm	0.599692**	0.292951**	0.182489**	0.956264	0.096832**	0.201986**	0.517523**	1.334322
MSGARCHnorm	0.599692**	0.292951**	0.082859**	0.956264	0.69809**	0.734075**	0.989647**	0.926612
GARCHstd	0.332944**	0.318135**	0.327701**	1.082283	0.102975**	0.231256**	0.864046**	0.704225
MSGARCHstd	0.592603**	0.140428**	0.077869**	1.045219	0.69809**	0.734075**	0.950101**	0.926612
GARCHged	0.538857**	0.293491**	0.336994**	0.948851	0.038264	0.104931**	0.666176**	0.630096
MSGARCHged	0.797141**	0.535237**	0.134944**	0.978503	0.69809**	0.452346**	0.734606**	0.926612
GARCHsnorm	0.797141**	0.099346**	0.215052**	0.978503	0.066456**	0.153107**	0.399228**	1.371386
MSGARCHsnorm	0.866417**	0.264959**	0.091541**	0.985915	0.996913**	0.761058**	0.915913**	1.000741
GARCHsstd	0.189745**	0.178477**	0.209194**	1.111935	0.102975**	0.231256**	0.875625**	0.704225
MSGARCHsstd	0.426311**	0.224706**	0.06562**	1.067457	0.848702**	0.762395**	0.176027**	0.963677
GARCHsged	0.538857**	0.113066**	0.218791**	0.948851	0.064408**	0.160255**	0.758104**	0.667161
MSGARCHsged	0.85319**	0.4361**	0.172491**	1.015567	0.319515**	0.508443**	0.853383**	0.815419
*Soybeans*								
GARCHnorm	0.070937**	0.074143**	0.339663**	0.852167	0.011746	0.022208	0.068071**	2
MSGARCHnorm	0.373825**	0.425806**	0.84635**	0.926269	0.996913**	0.761058**	0.644437**	1.000741
GARCHstd	0.793748**	0.777613**	0.962079**	0.97814	0.848702**	0.762395**	0.58281**	0.963677
MSGARCHstd	0.478429**	0.205038**	0.569573**	0.941089	0.556782**	0.678314**	0.636652**	0.889548
GARCHged	0.150896**	0.169852**	0.637644**	0.881808	0.429471**	0.60052**	0.876634**	0.852483
MSGARCHged	0.245318**	0.282074**	0.577385**	0.904039	0.848702**	0.762395**	0.859678**	0.963677
GARCHsnorm	0.010551	0.025755	0.251902**	0.792886	0.137853**	0.209911**	0.318147**	1.297257
MSGARCHsnorm	0.210175**	0.43667**	0.724079**	0.896628	0.228641**	0.410883**	0.898333**	0.778354
GARCHsstd	0.210175**	0.351503**	0.770771**	0.896628	0.429471**	0.60052**	0.463687**	0.852483
MSGARCHsstd	0.037009	0.061497**	0.309101**	0.829937	0.102975**	0.231256**	0.807443**	0.704225
GARCHsged	0.029299	0.072187**	0.42747**	0.822527	0.228641**	0.410883**	0.766017**	0.778354
MSGARCHsged	0.046355	0.045807	0.219669**	0.837347	0.319515**	0.508443**	0.808987**	0.815419
*Sugar*								
GARCHnorm	0.247085**	0.497879**	0.732411**	0.904374	0.096832**	0.201986**	0.033076	1.334322
MSGARCHnorm	0.127491**	0.268296**	0.767797**	0.874722	0.002628	0.010177	0.301284**	0.481838
GARCHstd	0.85319**	0.910519**	0.689048**	1.015567	0.319515**	0.240722**	0.096096**	0.815419
MSGARCHstd	0.729269**	0.542411**	0.456444**	0.97109	0.064408**	0.160255**	0.58733**	0.667161
GARCHged	0.211755**	0.449912**	0.560649**	0.896961	0.064408**	0.050112**	0.128026**	0.667161
MSGARCHged	0.127491**	0.268296**	0.090227**	0.874722	0.228641**	0.176721**	0.002815	0.778354
GARCHsnorm	0.376169**	0.635087**	0.664289**	0.926612	0.011746	0.037856	0.015267	1.519644
MSGARCHsnorm	0.936567**	0.960533**	0.02223	0.993328	0.102975**	0.080877**	0.028972	0.704225
GARCHsstd	0.592603**	0.745351**	0.606945**	1.045219	0.996913**	0.549507**	0.012688	1.000741
MSGARCHsstd	0.53389**	0.807748**	0.203627**	1.052632	0.848702**	0.509321**	0.23263**	0.963677
GARCHsged	0.376169**	0.673278**	0.767099**	0.926612	0.228641**	0.176721**	0.084522**	0.778354
MSGARCHsged	0.866417**	0.959651**	0.423016**	0.985915	0.429471**	0.311619**	0.137356**	0.852483
*Wheat*								
GARCHnorm	0.018101	0.046651	0.272935**	0.808006	0.228641**	0.410883**	0.29739**	0.778354
MSGARCHnorm	0.127491**	0.17592**	0.731896**	0.874722	0.064408**	0.160255**	0.781315**	0.667161
GARCHstd	0.211755**	0.366135**	0.43829**	0.896961	0.102975**	0.231256**	6.3e−05	0.704225
MSGARCHstd	0.152128**	0.300643**	0.533684**	0.882135	0.005665	0.020242	0.069853**	0.518903
GARCHged	0.023255	0.056412**	0.205177**	0.815419	0.038264	0.104931**	0.05844**	0.630096
MSGARCHged	0.152128**	0.193574**	0.630419**	0.882135	0.011397	0.037439	0.117407**	0.555967
GARCHsnorm	0.058145**	0.153375**	0.474211**	0.84507	0.844493**	0.73126**	0.633005**	1.037806
MSGARCHsnorm	0.329276**	0.456365**	0.880336**	0.919199	0.102975**	0.231256**	0.414457**	0.704225
GARCHsstd	0.538857**	0.740983**	0.577715**	0.948851	0.228641**	0.410883**	0.000269	0.778354
MSGARCHsstd	0.797141**	0.535237**	0.677215**	0.978503	0.102975**	0.231256**	0.402083**	0.704225
GARCHsged	0.106023**	0.236974**	0.457106**	0.867309	0.102975**	0.231256**	0.133795**	0.704225
MSGARCHsged	0.286243**	0.428291**	0.69054**	0.911787	0.038264	0.104931**	0.24896**	0.630096
*Soybean Oil*								
GARCHnorm	0.247085**	0.01933	0.068365**	0.904374	0.260377**	0.098552**	0	1.223128
MSGARCHnorm	0.538857**	0.061719**	0.129585**	0.948851	0.447366**	0.112431**	2.00E−06	1.148999
GARCHstd	0.376169**	0.036285	0.069837**	0.926612	0.848702**	0.078492**	0	0.963677
MSGARCHstd	0.6633**	0.037262	0.0381	0.963677	0.844493**	0.102753**	0	1.037806
GARCHged	0.211755**	0.015324	0.054546**	0.896961	0.69809**	0.064088**	0	0.926612
MSGARCHged	0.180153**	0.012011	0.046329	0.889548	0.996913**	0.09181**	0	1.000741
GARCHsnorm	0.481144**	0.022126	0.060051**	0.941438	0.066456**	0.049959	0	1.371386
MSGARCHsnorm	0.922692**	0.035904	0.08193**	1.008154	0.029222	0.02937	1.00E−06	1.445515
GARCHsstd	0.992953**	0.031922	0.043938	1.000741	0.699371**	0.11023**	0	1.07487
MSGARCHsstd	0.592603**	0.055455**	0.043967	1.045219	0.447366**	0.112431**	0	1.148999
GARCHsged	0.286243**	0.024111	0.087524**	0.911787	0.996913**	0.09181**	0	1.000741
MSGARCHsged	0.992953**	0.07185**	0.073024**	1.000741	0.137853**	0.012129	0	1.297257
*Orange Juice*								
GARCHnorm	0.784965**	0.046657	0.006078	1.02298	0.000131	0.000664	2.00E−06	1.81616
MSGARCHnorm	0.53389**	0.221842**	0.003341	1.052632	0.848702**	0.762395**	0.197053**	0.963677
GARCHstd	0.08253**	0.030287	0.001254	1.148999	0.429471**	0.60052**	0.29819**	0.852483
MSGARCHstd	0.25423**	0.208511**	0.001421	1.097109	0.699371**	0.677201**	0.129704**	1.07487
GARCHged	0.654254**	0.024493	0.003877	1.037806	0.102975**	0.231256**	0.238843**	0.704225
MSGARCHged	0.922692**	0.038134	0.008726	1.008154	0.996913**	0.761058**	0.010889	1.000741
GARCHsnorm	0.784965**	0.046657	0.005183	1.02298	3.5e−05	0.000191	0	1.890289
MSGARCHsnorm	0.718505**	0.050828**	0.000302	1.030393	0.844493**	0.73126**	0.107508**	1.037806
GARCHsstd	0.068662**	0.028494	0.001259	1.156412	0.319515**	0.508443**	0.224408**	0.815419
MSGARCHsstd	0.53389**	0.061884**	0.000893	1.052632	0.844493**	0.73126**	0.015246	1.037806
GARCHsged	0.784965**	0.020155	0.001836	1.02298	0.156918**	0.316237**	0.273965**	0.74129
MSGARCHsged	0.654254**	0.024493	0.001054	1.037806	0.565949**	0.605186**	0.007519	1.111935
*Soybean Meal*								
GARCHnorm	6.00E−05	0.00032	0.011323	0.681987	0.260377**	0.352689**	0.001867	1.223128
MSGARCHnorm	0.0024	0.009907	0.066647**	0.755835	0.996913**	0.761058**	0.020671	1.000741
GARCHstd	0.003333	0.011939	0.094688**	0.763529	0.156918**	0.316237**	0.007041	0.74129
MSGARCHstd	0.004522	0.015539	0.123757**	0.770941	0.319515**	0.508443**	0.010816	0.815419
GARCHged	4.00E−06	2.3e−05	0.002788	0.637509	0.038264	0.104931**	0.00335	0.630096
MSGARCHged	0.000204	0.000995	0.0244	0.704225	0.556782**	0.678314**	0.031888	0.889548
GARCHsnorm	4.00E−06	2.3e−05	0.002749	0.637509	0.699371**	0.677201**	0.004273	1.07487
MSGARCHsnorm	0.003288	0.011234	0.0703**	0.763246	0.69809**	0.734075**	0.004694	0.926612
GARCHsstd	0.003333	0.011939	0.096203**	0.763529	0.319515**	0.508443**	0.005971	0.815419
MSGARCHsstd	0.023255	0.074293**	0.256971**	0.815419	0.69809**	0.734075**	0.007531	0.926612
GARCHsged	3.9e−05**	0.000214	0.011576	0.674574	0.156918**	0.316237**	0.002567	0.74129
MSGARCHsged	0.003333	0.013443	0.11186**	0.763529	0.556782**	0.678314**	0.007598	0.889548

We report the *p* values of the Unconditional Coverage (uc.LRp), Conditional Coverage (cc.LRp), Dynamic Quantile (DQp) tests and the Actual over Expected Exceedances Ratio (AE) computed at the 95% and 99% confidence levels. The models included in the assessment are GARCH(1,1) with innovations following a Normal (GARCHnorm), Skewed Normal (GARCHsnorm), Student’s-t (GARCHstd), Skewed Student’s-t (GARCHsstd), Generalized Error Distribution (GARCHged), and Skewed Generalized Error Distribution (GARCHsged) and MS-GARCH(1,1) following a Normal (MSGARCHnorm), Skewed Normal (MSGARCHsnorm), Student’s-t (MSGARCHstd), Skewed Student’s-t (MSGARCHsstd), Generalized Error Distribution (MSGARCHged), and Skewed Generalized Error Distribution (MSGARCHsged)

* Denotes the *p* values higher than the 5% significance level

** Denotes the *p* values higher than the 1% significance level

Sample period: October 3, 2005–March 25, 2022

**Table 8:** Degree of connectedness between each commodity and the three commodity sectors

	Metals	Energy	Agriculture
Gold	0.75	0.14	0.25
Silver	1.00	0.71	0.67
Palladium	1.00	0.43	0.75
Copper	1.00	0.57	0.67
Zinc	0.75	0.14	0.67
Heating Oil	0.20	0.67	0.25
Low Sulfur Gasolio	0.80	1.00	0.58
Gasoline	0.60	0.83	0.50
Natural Gas	0.00	1.00	0.58
Ethanol	0.40	0.67	0.67
WTI Crude Oil	0.60	1.00	0.58
Natural Gas UK	0.20	0.50	0.17
Oats	0.80	0.29	0.82
Wheat	0.00	0.29	0.82
Cocoa	0.80	0.57	0.36
Corn	0.40	0.43	0.82
Cotton	1.00	0.43	1.00
Sugar	0.80	0.43	0.73
Soybean Oil	0.80	1.00	0.91
Soybean Meal	0.00	0.43	0.82
Orange Juice	0.60	0.57	0.64
Coffee	0.80	0.43	0.91
Soybeans	0.60	0.14	0.91
Rough Rice	0.60	0.71	0.73

Sample period: October 3, 2005–March 25, 2022

**Table 9:** Degree of connectedness between the commodity sectors

	Metals	Energy	Agriculture
Metals	0.90	0.40	0.60
Energy	0.40	0.81	0.48
Agriculture	0.60	0.48	0.79

Sample period: October 3, 2005–March 25, 2022

**Table 10:** Cluster identification of the commodities in the sample in Fig. 2

	Cluster analysis
Gold	1
Silver	1
Palladium	1
Copper	1
Zinc	1
Heating Oil	3
Low Sulfur Gasolio	3
Gasoline	3
Natural Gas	3
Ethanol	2
WTI Crude Oil	3
Natural Gas UK	3
Oats	2
Wheat	2
Cocoa	1
Corn	2
Cotton	2
Sugar	2
Soybean Oil	2
Soybean Meal	2
Orange Juice	3
Coffee	2
Soybeans	2
Rough Rice	2

Number 1 denotes the red cluster, number 2 the green cluster, and number 3 the purple cluster. Sample period: October 3, 2005–March 25, 2022

## Footnotes

1 We refer to Table 4 in the Appendix for a global summary of the works on commodity markets.

2 Since daily returns are generally small in monetary units, they have been multiplied by 100 to avoid numerical errors in computer programs.

3 The official arbiter of U.S. recessions.
